# Barriers and enablers to nurse practitioner implementation of medication abortion in Canada: A qualitative study

**DOI:** 10.1371/journal.pone.0280757

**Published:** 2023-01-26

**Authors:** Andrea Carson, Emma Stirling-Cameron, Martha Paynter, Sarah Munro, Wendy V. Norman, Kelley Kilpatrick, Stephanie Begun, Ruth Martin-Misener

**Affiliations:** 1 School of Nursing, Dalhousie University, Halifax, Nova Scotia, Canada; 2 Faculty of Health, Dalhousie University, Halifax, Nova Scotia, Canada; 3 Department of Obstetrics and Gynaecology, University of British Columbia, Vancouver, British Columbia, Canada; 4 Department of Family Practice, University of British Columbia, Vancouver, British Columbia, Canada; 5 Faculty of Public Health & Policy, London School of Hygiene & Tropical Medicine, London, United Kingdom; 6 Ingram School of Nursing, McGill University, Montreal, Quebec, Canada; 7 Factor-Inwentash Faculty of Social Work, University of Toronto, Toronto, Ontario, Canada; University of Bedfordshire, UNITED KINGDOM

## Abstract

In this study we explored nurse practitioner-provided medication abortion in Canada and identified barriers and enablers to uptake and implementation. Between 2020–2021, we conducted 43 semi-structured interviews with 20 healthcare stakeholders and 23 nurse practitioners who both provided and did not provide medication abortion. Data were analyzed using interpretive description. We identified five overarching themes: 1) Access and use of ultrasound for gestational dating; 2) Advertising and anonymity of services; 3) Abortion as specialized or primary care; 4) Location and proximity to services; and 5) Education, mentorship, and peer support. Under certain conditions, ultrasound is not required for medication abortion, supporting nurse practitioner provision in the absence of access to this technology. Nurse practitioners felt a conflict between wanting to advertise their abortion services while also protecting their anonymity and that of their patients. Some nurse practitioners perceived medication abortion to be a low-resource, easy-to-provide service, while some not providing medication abortion continued to refer patients to specialized clinics. Some participants in rural areas felt unable to provide this service because they were too far from emergency services in the event of complications. Most nurse practitioners did not have any training in abortion care during their education and desired the support of a mentor experienced in abortion provision. Addressing factors that influence nurse practitioner provision of medication abortion will help to broaden access. Nurse practitioners are well-suited to provide medication abortion care but face multiple ongoing barriers to provision. We recommend the integration of medication abortion training into nurse practitioner education. Further, widespread communication from nursing organizations could inform nurse practitioners that medication abortion is within their scope of practice and facilitate public outreach campaigns to inform the public that this service exists and can be provided by nurse practitioners.

## Introduction

Access to safe abortion is a fundamental human right and critical component of comprehensive sexual and reproductive healthcare [1]. Medication abortion (MA) is a safe and effective method for pregnancy termination [2]. The drug combination using the pills mifepristone (200 mg), causing breakdown in the placenta, and misoprostol (800 μ), causing expulsion of the pregnancy, is the international gold standard for first trimester MA [3] and is legally available in approximately 60 countries worldwide [4]. MA with mifepristone/misoprostol accounts for up to 60% of all abortions in some European countries [5] and 54% in the United States [6]. Data show that women and pregnant people who choose MA are satisfied with the process, with 63% to 96% of users indicating that they would opt for MA again [3]. Preference for MA was attributed to privacy, convenience, lack of surgery and/or anesthesia, and increased accessibility for patients in rural and remote communities [7, 8].

While abortion was fully decriminalized in Canada in 1988 [9], the federal drug regulator, Health Canada, did not approve mifepristone until 2015 [10]. In January 2017, the drug became available on the Canadian market with several restrictions including: physician-only prescribing; physician dispensing of mifepristone; and mandatory ultrasound for gestational dating and pregnancy location [10]. Clinicians, researchers, legal experts, and patients pushed back against the restrictions, as they created unnecessary barriers to access and potential harms to patients [11, 12]. In November 2017, these restrictions on the drug were removed thereby allowing non-physician healthcare providers to prescribe and dispense mifepristone [13]. These regulatory changes arguably increased uptake from fewer than 3% of abortions using other medications prior to mifepristone: in the two years after the introduction and deregulation of mifepristone, 31.4% of abortions conducted in Canada’s largest province (Ontario) were medical [2].

Nurses have a strong tradition providing and leading reproductive health and abortion care [14]. They play key roles including the provision of abortion counselling and decision-making support, dispensing abortifacient agents, contraceptive counselling, and, in some countries, the provision of medical and surgical abortions [15]. Yet nurses remain under-utilized members of abortion care teams possibly because their nurse practitioner (NP) education programs did not include abortion [16]. Systematic reviews of randomized controlled trials found no significant differences between formally trained provider groups (nurses, midwives, physician assistants) and physicians providing surgical and medical termination of pregnancy [17–19]. Expanding nurse involvement in and autonomy over abortion care could improve access to and use of safe abortion services [20].

Canada has made critical strides towards advancing nurse practitioners’ independent provision of MA care [13]. MA is within the legal scope of practice of nurse practitioners (NPs) across all of Canada’s 10 provinces and 3 territories [21]. NPs are registered nurses who have completed additional graduate-level education and training [22]. NPs have a broad legal scope of practice that includes the ability to autonomously diagnose and treat illnesses, order tests, prescribe medication, and perform certain medical procedures [22]. As of 2020, there were 6,661 NPs licensed to practice across Canada [21]. The majority of NPs work in community and hospital settings [21] and help facilitate access to care for people in rural and remote communities [23]. The total NPs currently providing MA in Canada is unknown. NPs are largely paid salaries, without billing codes that would indicate the service provided. For comparison, most physicians in Canada operate on a fee-for-service basis and have billing codes associated with medication abortion care.

Despite the complete decriminalization of abortion in Canada, outstanding barriers to access remain [24]. Barriers persisted for providers and potential providers even after the removal of many MA restrictions in 2017 [25]. These barriers include difficulties navigating the changing policy guidelines governing mifepristone prescription; fear of stigma or harassment; and patient proximity to services [25]. Some barriers are unique to MA. Although primary care providers are authorized to prescribe MA, many continue to refer patients out of their clinics for services [25]. In Australia, some primary care providers declined to incorporate MA in their practice because they perceived it to be a specialized service, and believed they were not located close enough to emergency and surgical services in the event of complications [26]. This may also be the case for NPs in Canada. Most NPs in Canada have not received any formal education around abortion care as part of their graduate training [27]. NPs themselves may be unaware that they are able to provide, and similarly, the public may be unaware that NPs can independently prescribe MA [16].

The integration of MA into NP scope of practice in Canada is a unique opportunity to increase the accessibility of abortion care and normalize abortion as part of primary care. NP provision of MA may create more equitable access to abortion, particularly for underserved populations across Canada, by increasing the total number of providers of abortion, especially in rural and remote areas, and for a wider range of patients [23, 28]. Nurses remain the largest health provider group globally, yet few countries permit NPs to prescribe MA [29]. The findings from this paper are part of a larger multi-methods study, “CART Canadian NP Mifepristone Implementation Study,” the overarching aim of which was to explore the implementation of mifepristone for MA amongst NPs in Canada–including experiences of current NP providers and barriers to NP provision of MA–to generate insights that could inform the development of interventions to better support NPs to implement this service. This paper reports on the qualitative findings from this study, drawn from interviews with NPs and stakeholders about their experiences with MA implementation. Building on our qualitative findings about the value-add of NPs for abortion care using a feminist lens [30], here we focus specifically on identified barriers and enablers to NP provision of MA (e.g., resources, geography, health system considerations, communications, regulations, clinical dynamics).

## Methods

### Study design

The study design was informed by our previous Contraception and Abortion Research Team (CART) study on the barriers and facilitators to MA implementation for physicians and pharmacists [12]. We used qualitative methods to identify enablers and barriers to MA, according to stakeholders and NPs. Study instruments, including the interview guides, were developed iteratively by the research team and included engagement from clinical and qualitative experts from a variety of disciplines and social locations. An interpretive descriptive approach guided qualitative data collection and analysis [31]. Interpretive description takes a pragmatic approach with the goal of advancing clinical care, particularly in areas of nursing science [32, 33]. In alignment with this framework, we operate from the perspective that researchers are an integral part of the research process, analysis, and interpretation of findings [33]. We bring expertise in public health, nursing, clinical obstetrics and gynecological care, social sciences and sexual and reproductive health advocacy to our analysis. We approached this research as supporters of family planning, with the value that abortion care should be safe and accessible for all people in Canada. We apply a lens of health equity to understand how ongoing social and structural barriers impact the health of underserved populations, and how stigma associated with abortion continues to impact its integration and acceptability in Canada’s health system [34, 35].

### Participants

Two participant groups were recruited for this project: stakeholders and NPs.

The first included key stakeholders working in government, health administration (e.g., clinical directors), nursing regulation, and law. The second included NPs who had and had not provided MA.

### Data collection

Stakeholder participants were recruited through purposeful and snowball sampling [36, 37]. Potential stakeholders were invited to participate through networks of the research team and knowledge users. Additionally, we contacted nursing organizations in Canada (e.g., provincial nursing associations, Canadian Nurses Association) and sexual and reproductive health clinics (e.g., Planned Parenthood) to invite stakeholders to participate in interviews.

We used purposeful sampling [36, 38] to select NP participants from respondents of our national survey of NPs who provided and did not provide MA (manuscript in preparation). The survey was distributed through the member networks of the following organizations: British Columbia Nurse Practitioners Association; L’Association des infirmières praticiennes spécialisées du Québec (AIPSQ); National Abortion Federation of Canada; Newfoundland and Labrador Nurse Practitioners Association; Nurse Practitioners Association of Alberta; Nurse Practitioners Association of Canada; Nurse Practitioners Association of Ontario; Nurse Practitioners Association of Manitoba; Nurses Association of New Brunswick; Nurse Practitioners Association of Nova Scotia; Prince Edward Island Nurse Practitioners Association; Saskatchewan Association of Nurse Practitioners. Additionally, the survey was distributed amongst NP members of the Canadian Abortion Providers Support (CAPS) Network (https://caps-cpca.ubc.ca/index.php?title=Main_Page).

During the consent process, survey respondents indicated whether they would be willing to be contacted for an interview. NPs were recruited to include diversity with respect to rural/remote or urban settings, region of Canada, and practice setting (e.g. primary care, sexual/reproductive healthcare). Few NP respondents that did not provide MA indicated they were willing to participate in a qualitative interview. Due to the size of the group and particular interest in reasons for not providing MA, all non-providing NPs who agreed to be contacted for an interview were invited to participate. All participants signed an informed consent document explaining the study and confidentiality.

All interviews were conducted remotely via telephone or videoconferencing by members of the research team trained in qualitative interviewing methods (AC, ESC, MP, KK). Interviews ranged from 30 to 60 minutes in length, and were offered in French and English. Honoraria were not provided. Monthly meetings were held with study co-investigators to discuss and revise the interview guide. The interview guide included questions about the NP’s practice context and barriers and enablers to provision of MA. Sample questions included: How did you become involved in providing medication abortion? What was your experience like bringing medication abortion into your practice? What are some of the benefits of implementing medication abortion into your practice? How has your provision of medication abortion impacted your patients? What is the value of having NPs as abortion providers in Canada?

### Analysis

All interviews were digitally recorded and transcribed verbatim and translated into English (if completed in French) by a professional medical transcriptionist and French-language translator. Two members of the research team (AC, ESC) independently read each transcript and wrote memo notes in the margins. Following initial reading and memo notetaking, authors AC and ESC independently coded transcripts into common categories based on topic/area (e.g. practice setting, clinical description, care process, challenges to provision, opportunities and supports), after which joint meetings were held to discuss the content of each transcript. A codebook was developed based on consensus between the two coders and the co-PI (RMM), which was then used to code (categorize) remaining transcripts. Throughout data collection, members of the research team met weekly to debrief the interviews and discuss commonalities, contradictions, and areas for further exploration. Advice and clinical insights were offered by two nurse-scientists (RMM, MP) and another clinician member of our team (WVN). When the researchers were hearing similar ideas, with few new concepts or issues being identified, we had discussions with the core team before concluding there was enough rich data to analyze and conduct a rigorous interpretation of data that met our objectives.

### Ethics

Ethics approval was received from Nova Scotia Health Research Ethics Board and the University of British Columbia Research Ethics Board in 2019. Participants were emailed an informed consent document prior to participation. Participants were assigned pseudonyms and contextual details (role title, province) were separated from responses for de-identification.

## Findings

Forty-three interviews were conducted with stakeholders (n = 20) and NPs (n = 23) across Canada. Stakeholders represented areas of health administration, government, nursing regulation, and sexual and reproductive health advocacy at national and provincial/territorial levels. Of the NPs, 16 provided MA and seven were not providing MA at the time of participation. NPs worked in NP-led clinics, sexual and reproductive health clinics, and primary care clinics. Twenty NPs self-identified as women and three as men. Eight NPs were from Ontario, three from New Brunswick, three from British Columbia, two from Saskatchewan, two from Manitoba, one from Alberta, one from Quebec, and one from the Yukon.

We organized the findings into five themes which reflect the practice, health systems, and individual factors related to implementation of MA: (1) use of ultrasound for gestational dating; (2) abortion as specialized or primary care (3) advertising and provider anonymity; (4) geography and proximity to services; and (5) education, mentorship, and peer support. Below, we describe each theme in detail and provide an overview of these barriers and enablers to MA provision in Table 1.

**Table 1:** Summary of key enablers and barriers to NP provision of medication abortion in Canada.

	Enabler to MA provision	Barrier to MA provision
**Ultrasound**	There is no legislative requirement to conduct ultrasound prior to prescription. This was an enabler to provision for several NPs.	Access to timely ultrasound and comfort level/confidence to provide MA without ultrasound was a barrier for some NPs.
**Advertising**	In primary care, being able to advertise MA amongst a range of other services, was a benefit for some NPs because it normalizes MA within a range of sexual and reproductive health services.	A barrier for NPs advertising MA was the perception of potential stigma. This reduces patient knowledge about who offers MA and may prevent providers from connecting with others who offer this service.
**Specialized care**	Specialized reproductive and sexual health clinics provided support to NPs and their patients around abortion care; a place to connect services and share resources about MA.	The perception that abortion is a specialized service may prevent eligible providers from implementing it in their practices. Where specialized clinics were well-established and offered abortions, some NPs opted to refer patients rather than implement MA in their own practice.
**Infrastructure and resources**	NPs in urban areas with access to resources (24/7 emergency services, timely ultrasound when needed, pharmacies) had success implementing MA.	NPs in rural and remote areas had concerns about implementing MA in the absence of locally-based emergency services, labs to process bloodwork, and pharmacies willing to stock the medication. NPs in these areas largely did not provide MA.
**Education and mentorship**	Mentorship was highly valuable for NPs. Where mentorship in abortion care was available, NPs had high-level of confidence to provide and felt supported	Where NPs did not know other MA providers in their clinic or community, they felt isolated and that they could not deliver MA efficiently.

### Use of ultrasound

Ultrasound is not required in Canada prior to the provision of MA; however, it is recommended in clinical cases where gestational dating or diagnosis of ectopic pregnancy may be in doubt [3]. MA has a gradually lower effectiveness with each additional week of gestational age and becomes less effective than surgical abortion after nine weeks gestation. Although MA will not worsen an ectopic pregnancy, the early detection and management of this rare form of pregnancy is important due to the natural progression to severe bleeding or death without appropriate intervention [3].

Some NP participants used ultrasound regularly while others included ultrasound only on an as-needed basis (e.g., when a patient was unsure of the date of their last menstrual period or was at risk for an ectopic pregnancy). Factors affecting participants’ use of ultrasound included proximity and access to timely ultrasound; their comfort level dating a pregnancy without ultrasound (using date of last menstrual period, manual examination, and/or bloodwork); and personal beliefs around the necessity of ultrasound and patient comfort.

Providers with efficient access to ultrasound regularly included it as part of their MA practice. For example, Amanda’s clinic was connected to a radiology department that prioritized appointments for gestational dating for abortion. Knowing that patients had expedited access to ultrasound motivated them to use ultrasound every time when providing MA: “The ultrasound department prioritizes any dating ultrasounds; they’re usually done within 24 hours of our requisitions being received.” This provider, and others with timely, nearby access to diagnostic imaging, felt that there was no downside or risk in using the ultrasound to confirm gestational age and to ensure their intrauterine pregnancy.

Other providers used ultrasound on an as-needed basis due to long wait times or travel required to access ultrasound. Cindy (NP Provider) described the situation in their community, “I’m lucky to get an ultrasound within ten days and that’s with my receptionist phoning around, phoning around to seven different places to try to get the earliest ultrasound.” In these circumstances, participants recalled having to decide whether to prescribe without ultrasound or accept the delays. For NP providers who depend on ultrasound to provide MA, limited access to this resource was a critical barrier.

Yeah, if you’re working in an area where an ultrasound is not easily accessible and based on your assessment of the patient, it’s something you feel is necessary, then that would obviously be a barrier to providing the medical abortion at that time. It might then delay the provision of that care and the opportunity for that [patient] to benefit from the medical abortion.

Joanne, Stakeholder

All NP providers described the importance of the efficiency of the MA process for patients; the sooner the termination could be completed once a pregnant person decided they wanted MA and it was clinically appropriate, the better it was for patient comfort and health. As MA is only approved by Health Canada for prescription up to nine weeks gestation, a delay in access to ultrasound can limit patient options, as described by Amanda below.

Mifegymiso has such a short window, often people don’t find out they’re pregnant until seven or eight weeks, we can’t delay by a month waiting for an appointment time. Getting that fast turnaround as a provider is huge, to increase access, and to make sure that as many as possible of our clients are able to access [medication] abortion.

Amanda, NP Provider

Several providers preferred not to use ultrasound because they felt it was unnecessary and invasive for routine use. These participants felt it should only be used when indicated from a clinical and patient safety perspective.

It’s available, but it’s not essential. The guidelines say it’s not essential anymore and it shouldn’t be a barrier to providing. Again, women have enough intervention with their bodies, so I usually will, in the right clinical situation, offer that option.

Monica, NP Provider

This NP recognized the patient discomfort often associated with ultrasound use, and the frequency with which women’s reproductive organs are probed for the purpose of medical intervention. Several NPs emphasized that ultrasound use may not be necessary for patients who were well below the 9-week cut-off for MA use and were certain of the dates of their last menstrual period.

### Advertising and provider anonymity

When discussing public and/or patient knowledge about their services (including MA), NP providers in primary care settings frequently stated that they did not explicitly advertise their MA services. Several NPs described how information about the availability of MA was included within the range of sexual and reproductive health services offered on their clinic’s website. Others relied on patients to share knowledge about the availability of MA services via ‘word-of-mouth’. Many NPs (providers and non-providers), as well as stakeholders, talked about the delicate balance between advertising services so that people in the community know where to go for MA, and protecting the anonymity of providers. This was more of a concern for some NPs than others, and dependent on the culture of the communities they lived in. Some NPs perceived more community anti-choice backlash and potential violence, especially in smaller communities. Despite the apprehension around anti-choice protests or violence, almost no providers had experienced intimidation, aggression, or threat from the public because of advertising or providing services. As described in another paper from this study, the resistance that they did face, if any, came from other health professionals in their communities or clinics, or their employers [30].

Despite uncertainty about whether and how to advertise MA availability, NP providers in primary care believed that providing MA in their clinics added comfort and anonymity for their patients.

The other nice thing is because it’s a private family practice it’s nice for the patients because they’re not self-identifying as going to an abortion clinic. They’re just going to a clinic for an appointment. No one really knows why they’re there. The assumption is made that they’re just seeking out family practice care and because, we’re not secret, we definitely don’t keep it a secret that we’re doing [medication] abortions, but the only people that really know is the [abortion clinic] so it’s not like we’re being inundated with protestors or anything either. It does make a bit of a nicer experience, I think, for the patients as well.

Lisa, NP Provider

While the lack of advertising can be advantageous for patient anonymity and comfort, some felt it could come at a cost. For example, lack of explicit advertising and/or community outreach about MA service could leave some pregnant people ‘in the dark,’ unsure where to go to access services. One stakeholder described how problematic this could be, especially given the stigma that continues to persist around abortion care—without direct advertisement or knowledge sharing, patients in need may look for services elsewhere.

Unless you are a bit more intentional [about advertising and outreach], clients aren’t going to ask. Abortion is still such a stigmatized issue. [Providers] will say ‘well we just really don’t see that in our community’ and I’m like ‘yes you do’. One in three people are getting an abortion. Just because they’re not asking you doesn’t mean… that just means they’re not asking you. They’re getting it somewhere else and you haven’t created a space that makes your clients feel like they can talk to you about that.

Sophie, Stakeholder in health administration

Most NPs were keen to identify a solution that both improved access to MA while also maintaining anonymity and safety for patients and providers. A number of participants felt that there was value in keeping a central directory of providers who offer MA, as this both helped to connect patients to service providers, while also keeping the information of providers separate: “I’m sure that there are lots of pro-choice NPs and physicians out there who either maybe are providing [medication] abortion but it’s not something that they’re talking about or advertising.” Carrie, NP Provider. There were examples of provinces with toll-free self-referral lines which connects people seeking abortion to providers in the community. The directories are also a way for health professionals in the community to know where MA providers in their community were located. Without such directories or networking, there are limited ways for health professionals to know how to refer patients to abortion care.

### Abortion as specialized or primary care

It was common for NP participants who did not provide MA to refer patients to specialized abortion clinics. By specialized clinics, they were referring to clinics who solely offered sexual and reproductive health services. According to participants, these specialized clinics were set up to provide aspiration (surgical) abortion, and many now also offer MA. They perceived provision of MA to be burdensome to implement, or their employers were not supportive of implementation of MA from a resource perspective. However, there was variation between NPs who did not provide MA, as to their level of knowledge about MA and the amount of information, counselling, or follow-up they provided after a patient inquired about abortion and a referral was made to a specialized clinic. For some NPs, they described the efficiency of referring patients to a specialized clinic. The abortion clinics were perceived as well-organized and appropriately resourced to provide MA. In these cases, NPs felt confident in making referrals to these clinics. In another example, a participant worked in an NP-led primary care clinic and collaborated with a physician in the community as part of a shared-care model for abortion:

So it’s kind of shared care. We’re kind of like beginning and after, however we’re not [prescribing] the Mifegymiso and my colleague and I have some thoughts as to why we’re not doing that but there’s no restriction to us doing it but apparently the model we have serves us pretty well. We’re struggling to really find motivation to change it.

Ben, NP Non-Provider

Although this NP was not prescribing the medication, they were involved in several aspects of the abortion process. In many cases, however, NPs not providing MA had more limited knowledge and provided patients with fewer details about abortion services when referring them to specialized clinics: “If [patients] ask me for any information on different types [of abortion], I’d probably give it to them. I’ve never had that question from them. Most of them you just tell them you’re going to refer and they’re happy with that but definitely, I’m happy to give them resources on it.” Lousie, NP not providing MA.

Several NPs describe referring patients to abortion services that were located in different communities and required patients to travel to get there with no assistance from their clinic. “We refer to the women’s health unit here in rural [area] and we refer to the women’s health unit in [urban city] for that. Interviewer: Okay, that’s in a different community? P: Yeah. It’s 45 minutes away. It’s the closest major city.” Elaine, NP not providing MA. While some NPs refer to clinics that are close by and well-resourced to provide abortion care, others referred out of their communities–mainly rural–rather than implement MA in their own clinics.

NP providers found that they could provide abortion more efficiently by offering it in primary care. Pamela, NP Provider, stated that while other providers or clinics in the community sent their patients to the specialized clinic in the area, they decided to offer these services “in house,” which was beneficial:

It’s been something that has been fulfilling. Not that many clinics in [my city] provide this care, we do have an abortion clinic, that’s really great and so it’s kind of been like ‘well you just send everyone there because they now do [medication] abortions’. People are like ‘why put in all this work and potential if it’s liability or risk or whatever when you can just send them somewhere else’. But we have seen the direct benefits of providing that care in house and it hasn’t been as complex as I think a lot of people thought it might be, it’s just one less barrier.

Pamela, NP Provider

Several participants echoed this description but emphasized that non-providing NPs may think it is more complicated than it is to implement MA and say it is “easy” and “once you’ve done it once, it’s super easy.” Cindy, NP Provider.

Quebec is a unique case, in that it is the province that provides the highest proportion of surgical abortions in Canada [39]. We had great difficulty recruiting NPs (both providers and non-providers of MA) in Quebec. Our survey data (reported elsewhere) indicated that no NP respondents from Quebec were providing MA. Interview data from three Quebec participants indicated that there is still much confusion around MA provision in the province, which is likely affecting uptake of MA among NPs. One NP from Quebec indicated that they do not provide MA because the province does not allow them to do so. Two stakeholder participants emphasized that there were no legal restrictions preventing NPs from providing MA. They did however indicate that it may be related to training requirements from professional regulators in the province which could be burdensome for NPs.

### Geography and proximity to services

NPs and stakeholders discussed the potential for MA to improve access to abortion closer to home, in rural and remote areas. As one NP put it, “yeah, I think, you know, hopefully [medication] abortion will make a difference for people who have less ready access to the bigger centers.” (Carrie, NP Provider). Additionally, there were some NPs that were using virtual care in their provision of MA, which they described as increasing during COVID-19. This allowed some providers to reach a broader group of patients further from their clinics. However, most NPs were still doing the majority of abortion care in-person.

The optimism about the potential for abortion to become more accessible for people in rural and remote areas is challenged by ongoing resources issues, a lack of health professionals in the local community, and perceived stigma in some smaller communities. For NPs in rural and remote communities, it may not be feasible to offer MA due to a lack of access to ultrasound services, proximity to emergency services, and a lack of pharmacies with a regular stock of mifepristone. For NPs with inadequate resources or infrastructure, referring patients to clinics outside their communities was a common practice. One rural non-provider described the conflict patients face when seeking healthcare outside of their community:

I think it’s very difficult for women, especially in those communities and young women often have multiple children and they, I think, are really conflicted when they have to go out…they are very conflicted and don’t like to go until the last minute and sometimes we don’t get them out in time because they have children at home and they’re needed in the homes.

Diana, NP not providing MA

The burden of travel is unequal: those with children, inflexible workplaces, and lack of sick pay, face additional financial and logistical hurdles. Acknowledging the impact of travel and time away from home, one clinic had taken steps to prescribe and dispense mifepristone/misoprostol for MA in one appointment. This is possible by having a physician trained in point-of-care ultrasound and keeping a supply of mifepristone in-house.

We try recognizing, especially for some women, travel in from their communities is really problematic. We have one community six hours away so if women do have to travel in, then making it just be a one stop, one day kind of appointment and then they can follow up with the nursing station in the community [for blood work or complications]. Personally, we work hard to make it low barrier and not resource heavy.

Stacy, NP Provider

The presence of interprofessional teams or other health professionals locally who can support MA care in rural and remote communities was discussed by participants as a factor in their decision to provide or not provide abortion and contributed to how supported or isolated they felt.

I think the process that I use in the context of my community, in my office, works pretty well, even if I’m alone. I don’t have a nurse or other support that maybe you’d have in clinics that have a whole interdisciplinary team. Obviously that would be ideal; I would prefer not to have to work in what I call a silo, but instead have a variety of professionals who really offer better care for the patient.

Chloe, NP Provider

Finding support from healthcare providers for MA locally may be difficult because there are fewer healthcare providers overall in rural and remote areas, they may not advertise they offer/dispense MA or resources associated—either publicly or privately—and the stigma around abortion provision may be greater in rural or remote areas. According to one stakeholder, “some pharmacies in some small communities may not be supportive of abortion so then a [pregnant person] can’t access [MA] in that community” (Caroline, Stakeholder). There may be anti-abortion sentiment that leads to stigma and hesitancy around providing/dispensing/advertising MA. Or the social and personal repercussions of being an abortion provider in a smaller community may be greater where community members are more familiar with each other. As such, identifying mentors and peer support may be a challenge in these cases.

### Education, mentorship, and peer support

Almost all NPs surveyed stated that they received no formal training on abortion care during their RN or NP training. As a result, NPs were not initially aware of their potential to provide abortion care and what provision would include. Elaine, NP Non-Provider, described the limited amount of information that was offered during the NP program about women’s health in general, let alone abortion-specific care: “I don’t know if there was any. There was very little on women’s health at all. It was just a really quick [overview] so specifically about abortion, no.” Similarly, Lisa, NP Provider, stated that because NPs receive limited education on sexual and reproductive health, they were motivated to be a mentor / preceptor to students interested in learning more:

It doesn’t have to be huge portion on the Mife but just enough that students can know that it’s out there, know that they can seek out doing extra things on top of their primary care work. I think would be important and contraception care is terrible in NP school too, because I take students all the time. I know what their knowledge level is like and I do IUD contraception workshops with the students and their knowledge on contraception is poor too so if it’s poor on that, it’s probably going to be poor on [medication] abortion as well or just abortion in general.

One stakeholder said integrating MA prescribing into NP education is one of their “pie in the sky dreams” and would “make a huge difference in terms of access” and would be a “game-changer.” Zoe, stakeholder in sexual health advocacy.

Most NP providers completed the online MA training program offered by the Society of Obstetricians and Gynecologists of Canada (SOGC). While many NPs found this course to be a good baseline of information, there was a consensus that further in-vivo support was needed to work through particular cases in their practices. As Kevin, NP Provider, described: “It gave all the information but again theoretically it doesn’t really hit home as much until you go through the process a few times and then start to see the theoretical adverse events… A lot of it came with just a learned knowledge through the process as well.”

Several NP providers described receiving support and mentorship in abortion care either from an NP, physician, or obstetrician-gynecologist. NPs felt motivated to provide MA when they had colleagues in their clinics who already prescribed and could mentor them. Mentors provided case-specific advice, shared protocols, and coached providers through new experiences with MA. This was an invaluable support, as Amber, NP Provider, described, to boost confidence:

Yeah, [my mentor] was definitely very, very helpful and just being sure about those little cases because there’s always things that aren’t cookie cutter, right? You’ll get these results that are a little bit weird or a delayed response or just having someone to talk to and ask ‘what do I do in this case or am I on the right track?’ That’s really, really helpful. It [gave] me confidence to do that treatment.

NP’s in this study both providing and not providing MA said that having a formal network to identify mentors/mentees in their communities would be beneficial because this was not something for which most had access:

If there was a coordinated mentorship program, that would have been helpful. I felt very unprepared when I went out into practice, and I was like I don’t know what to do and I would call a physician colleague of mine who was also prescribing Mifegymiso but the two of us didn’t have a ton of experience.

Amanda, NP Provider

Some NPs discussed the potential for NP curriculum to incorporate abortion-specific education going forward. For most participants, at the time they completed their graduate nursing education, NPs were not permitted to prescribe MA. In this study, mentorship and working through specific case-scenarios was timely for participants who had completed training.

## Discussion

Decriminalizing abortion and expanding health professional scopes of practice are significant steps toward normalizing abortion and making it more accessible. However, policy and regulatory changes alone do not lead to accessible care. We found that there are multiple intersecting barriers that limit or prevent NPs uptake of MA in Canada, despite decriminalization of abortion (since 1988) and the addition of MA to their scopes of practice (since 2017). For NP’s not providing MA, contributors to their decision not to provide included lack of access to resources/infrastructure in the community (e.g., ultrasound, emergency services), lack of support from employers or colleagues, and perceived adequate proximity to specialized clinics offering abortions (i.e. referring patients out). For NP’s providing MA, several factors supported their provision of MA and continued success with implementation, including supportive colleagues and mentors, access to resources to support MA in their communities, and interprofessional collaborations for shared care. Taking lessons from current challenges and factors which facilitate NP provision of abortion care may inform steps for addressing implementation challenges within and beyond Canada.

We found that it was easier for some NPs to incorporate abortion care into their practices than others. For those that were providing, and in a way that they were largely satisfied with (no major challenges to provision), they described having supportive pro-choice mentors or colleagues in their practices or communities to whom they could share knowledge, resources, and questions. As described by Lee et al., (2022), stigma and moral opposition to abortion creates interprofessional tension that may make it more difficult for providers to implement evidence-based protocols in their practices, especially when this moral opposition comes from those in leadership positions [40]. In a companion qualitative paper for this study, we found that leadership and support from pro-choice NPs was critical for new providers of MA to learn and implement this service: this included informal training, relational support, and sharing of resources such as consent forms and other tools or documents [30]. Settings that included interprofessional collaboration (e.g., social workers, nurses, physicians) facilitated efficient, well-rounded abortion care for patients in cases where colleagues and/or employers supported this service and its delivery.

Ultrasound for gestational dating is not required to prescribe MA. We found that the integration of ultrasound in NP abortion practice varied and depended on provider preference and resources. Some NPs used ultrasound for most of their MA patients to rule out ectopic pregnancy and/or to confirm gestational dates. These were also typically NPs with easy access to ultrasound technology (e.g., close to the clinic, quick appointment times). Other NPs described taking a trauma-informed approach to their abortion care and believed that reducing the number of tests/procedures that pregnant people undergo was important, as well as reducing harm to patients that may result from a lengthy wait or travel time for ultrasound where resources were limited [30]. In such cases, NPs described ordering ultrasound only where appropriate (e.g., patient unsure of last period, potential for ectopic pregnancy). Clinical scope and professional “territory” issues have been noted elsewhere in a study in the United States [40], wherein primary care providers reported tensions with OB/GYN and radiology departments when proposing evidence-based ultrasound-as-needed protocols. While we noted some professional tensions amongst radiology technicians and NPs over NP authority, which delayed ultrasounds in some cases [30], we did not find there were major tensions between OB/GBYNs and radiology over the use of ultrasound for MA. The more pertinent issue seemed to be access to quick ultrasounds locally in cases where it was deemed necessary.

A lack of local infrastructure continues to impact NP provision of MA [39, 41]. While there is optimism that the availability of MA has improved abortion access in rural/remote areas by allowing more providers to offer the service, providing in rural/remote areas has not been easy because of resource and collaboration restraints. There are many rural and remote communities in Canada without 24-hour local emergency services. This was a major contributor to the decision for some NPs not to provide MA altogether. This points to a disconnect between the policy level and the implementation of MA in NP practice.

The hesitancy amongst some providers not to advertise abortion services is unsurprising given the continued stigmatization of abortion in Canada and potential for anti-choice protests or violence toward providers [42]. Physicians and NPs may be providers of abortion while being hesitant to communicate and advertise these services within their communities avoid stigma and potential scrutiny from colleagues, other health professionals, friends and family [25]. This limited advertising about abortion services facilitates a disconnect between provider knowledge and public awareness of MA services in their communities. To optimize MA in Canada, the public needs to know how to access abortion services from primary care clinics and that it is now within NP scope of practice to provide.

We found that NPs valued MA for the potential benefit of privacy for patients when offered in primary care or in sexual health clinics, amongst a range of other services. Previous research shows that patients are satisfied and would prefer abortion care in primary care settings [43, 44]. In a qualitative study of 15 women who received abortion care in family medicine settings, Summit et al. (2016) found that while participants were surprised to learn this service was offered at their clinics, they were highly satisfied and found that receiving care from a clinic and provider they knew created a context of trust and privacy [45]. Additionally, patients appreciated the privacy afforded within a primary care setting, its convenience and continuity.

Generally, NPs not providing MA were less knowledgeable about MA in terms of current regulations, the resources needed to provide MA, clinical protocols, and drug pharmacology.

Similar to previous studies about the implementation of MA in health professional practice, lack of support from employers or colleagues to provide abortion was an ongoing barrier for those who were not providing MA, and even for some providers was a source of frustration and something to work around [39]. The majority of providers, however, described supportive colleagues and local networks of health professionals including pharmacists and found these supports improved efficiency in care delivery [25, 46].

We propose several recommendations to better support NPs in Canada work to their full scope in abortion care. First, educators should take steps to integrate the full range of sexual and reproductive health services into NP curricula [16]. This may include practicum opportunities in addition to classroom learning. To build on this education, national and provincial nursing organizations and local networks could establish interprofessional MA mentorship networks. As we found, mentorship was a key contributor to successful practice and confidence of NPs as well as the ability to communicate with fellow abortion providers in their communities. Secondly, professional associations and regulators should make efforts to communicate to registered NPs that MA is within their scope to encourage provision and address any misconceptions about scopes of practice and resource requirements, which we found was confusing to some and varied by province/territory. Lastly, stakeholders in public education/health/outreach are fundamental to increasing community knowledge about the existence of MA and that this service may be available to them locally in primary care. Providers also have a responsibility to make their full range of services known to patients. However, stigma about being an abortion provider contributes to hesitancy to advertise; being well-connected and supported by other MA providers and employers is one factor in addressing such hesitancy.

Importantly, Quebec was the only province where we were not able to identify a single NP providing this service within the broader study (for both survey and interviews). According to Guilbert et al. (2020), the uptake of MA in Quebec is slow due to “restrictive medical policies, [and] vested interests in surgical provision and administrative inertia” (p.190) [47]. In Quebec, legislative changes came into effect January 25, 2021, with the “Act to amend the Nurses Act and other provisions in order to facilitate access to health services” as part of Règlement sur les Infirmières Praticiennes Spécialisées [48]. The legislative changes rendered NPs autonomous and enabled NPs to provide more comprehensive services to patients, expanding their scope of practice. Such changes allow NPs to diagnose patients. Prior to January 2021, NPs were only permitted to give “diagnostic impressions” based on patients’ symptoms, which would need to be confirmed by a physician. NPs are now considered autonomous and no longer need to work in partnership with a physician. Our study was not able to capture whether these legislative changes are impacting NP provision of MA in Quebec. A further challenge for our study recruitment was the timing; recruitment began in January 2020 and continued to May 2021. Recruiting healthcare professionals during the COVID-19 pandemic required sensitivity and may have impacted those willing to participate given that many health professionals were overburdened during this time.

## Conclusion

We found that NPs and nursing stakeholders support NP provision of MA and believe they are well-suited to provide abortion care. Some NPs experienced ongoing barriers to providing MA, including inefficient access to ultrasound where needed, poor access (if any) to local emergency services for patients, isolation from other pro-choice health professionals or collaborators in the community, and stigma around abortion. These are barriers that may impact their decision to advertise MA as a service they offer. When the NPs in this study had local resources and infrastructure to support MA and were part of shared-care models or had relationships with other pro-choice providers in the community, including mentorship, they were more successful in implementing MA in their practices. Our recommendations are: nursing educators incorporate the full range of sexual and reproductive health services NPs provide into curricula, complemented by interprofessional mentorship networks for MA; professional associations and regulators regularly communicate with members to dispel misconceptions about scope and resource requirements; and stakeholders in public health and sexual/reproductive health communicate information to the public about the availability of MA in primary care and the providers who can offer it.
